# Cosmeceutical Potential of *Mitragyna speciosa* (Kratom): Anti-Adipogenic and Antioxidant Properties of Extracts and Mitragynine

**DOI:** 10.3390/molecules30214256

**Published:** 2025-10-31

**Authors:** Sudthiworarak Kaewchompoo, Prapapan Temkitthawon, Kalyarut Phumlek, Neti Waranuch, Ngamrayu Ngamdokmai, Kornkanok Ingkaninan

**Affiliations:** 1 Center of Excellence for Innovation in Chemistry, Research and Innovation Cluster for Natural Health Products, Faculty of Pharmaceutical Sciences, Naresuan University, Phitsanulok 65000, Thailand; puynoon.puynoonsd@gmail.com (S.K.); prapapantem@gmail.com (P.T.); kalyarut.ph@nu.ac.th (K.P.); 2 Center of Excellence for Innovation in Chemistry, Department of Pharmaceutical Technology, Faculty of Pharmaceutical Sciences, Naresuan University, Phitsanulok 65000, Thailand; netiw@nu.ac.th; 3 Cosmetics and Natural Products Research Center, Faculty of Pharmaceutical Sciences, Naresuan University, Phitsanulok 65000, Thailand; 4 Department of Pharmaceutical Sciences, Burapha University, Chonburi 20000, Thailand; ngamrayu@gmail.com; 5 Center of Excellence for Innovation in Chemistry, Department of Pharmaceutical Chemistry and Pharmacognosy, Faculty of Pharmaceutical Sciences, Naresuan University, Phitsanulok 65000, Thailand

**Keywords:** Kratom, mitragynine, anti-adipogenic activity, anti-oxidant activity, lipid accumulation, anti-cellulite agent

## Abstract

Kratom (*Mitragyna speciosa* (Korth.) Havil.) is a medicinal plant containing bioactive alkaloids, notably mitragynine and 7-hydroxymitragynine, which are psychoactive compounds with analgesic and stimulant properties. Due to safety concerns, the use of Kratom leaves and mitragynine in oral pharmaceutical products is restricted. Therefore, their potential as topical cosmeceutical agents merits further exploration. This study aimed to investigate the antioxidant and anti-adipogenic activities of Kratom ethanolic (Et-MS) and alkaloid-rich (Alk-MS) extracts, as well as purified mitragynine, to determine whether mitragynine is the major bioactive compound responsible for lipid reduction in 3T3-L1 adipocytes. The antioxidant properties were assessed using DPPH, ABTS, and FRAP assays, yielding EC_5__0_ values of 0.06 mg/mL, 0.29 mg/mL, and 55 g Fe^2+^/100 g for Et-MS, respectively. In comparison, ascorbic acid (positive control) showed a DPPH EC_5__0_ value of 0.002 mg/mL. Both Alk-MS and mitragynine significantly inhibited lipid accumulation in 3T3-L1 adipocytes by up to 50–70% at non-cytotoxic concentrations (≤25 µg/mL), as determined by Oil Red O staining. These findings provide preliminary in vitro evidence that phenolic constituents contribute to antioxidant capacity, while mitragynine is the principal anti-adipogenic constituent in Kratom extracts. Collectively, the results support the potential for further development of Kratom-derived extracts and mitragynine as plant-based candidates for topical or cosmeceutical applications targeting subcutaneous fat and oxidative skin damage.

## 1. Introduction

Kratom (*Mitragyna speciosa* (Korth.) Havil.) [1], a medicinal plant native to Southeast Asia [2], is traditionally used for its stimulant, analgesic [3,4], and opioid-like effects [5]. Its major alkaloid, mitragynine [6,7], has been extensively studied for its pharmacological activities, including analgesic, stimulant, and psychoactive properties [5]. However, due to growing concerns regarding its potential adverse effects when administered orally, especially at high doses or with prolonged use, the application of Kratom and its alkaloids in oral pharmaceutical products is limited by safety considerations [8,9].

Transdermal or topical application may offer a safer and more targeted alternative, avoiding first-pass metabolism and minimizing systemic toxicity. In this context, the cosmeceutical potential of Kratom has attracted increasing interest, particularly for its bioactive properties that may benefit skin health and subcutaneous adipose tissue regulation. While traditionally recognized for its neurological actions, emerging evidence suggests that Kratom and its constituents may also exhibit bioactivities relevant to skin care, anti-cellulite treatment, and fat metabolism [10].

Several studies have reported that plant-derived alkaloids and polyphenols can influence adipogenesis by modulating oxidative stress, inflammation, and lipid-regulating pathways [11,12]. Kratom extracts are known to contain diverse phytochemicals, including antioxidant compounds [13] such as polyphenols and alkaloids [12,14], which may contribute to the regulation of lipid accumulation and redox balance in adipose tissue. These mechanisms are critical for addressing cosmetic conditions such as cellulite and broader metabolic disorders like obesity [15,16,17]. Although previous studies have reported that Kratom extract can reduce lipid accumulation as demonstrated by Oil Red O staining [10], the specific effects of Kratom extract and mitragynine on adipocyte lipid storage, oxidative stress, and metabolic pathways remain largely unexplored, particularly in the context of topical or cosmetic applications.

This study aims to investigate, in vitro, the antioxidant and anti-adipogenic effects of Kratom extracts and mitragynine, with a focus on their potential relevance to cosmeceutical formulations. We hypothesize that Kratom extracts, particularly their alkaloidal components, may reduce lipid accumulation in 3T3-L1 adipocytes through the modulation of oxidative stress and adipogenic pathways. Although the precise molecular mechanisms remain to be elucidated, such effects may involve redox-mediated regulation of adipocyte differentiation processes. To test this, ethanolic and alkaloid-rich extracts were prepared from Kratom leaves and evaluated for antioxidant activity and effects on lipid storage using Oil Red O staining. The findings from this study provide preliminary in vitro evidence supporting the potential of Kratom-derived compounds as natural anti-cellulite agents and their future development in transdermal cosmeceuticals targeting subcutaneous fat and improving skin texture.

## 2. Results and Discussion

### 2.1. HPLC Analysis and Mitragynine Content

**Figure 1:**
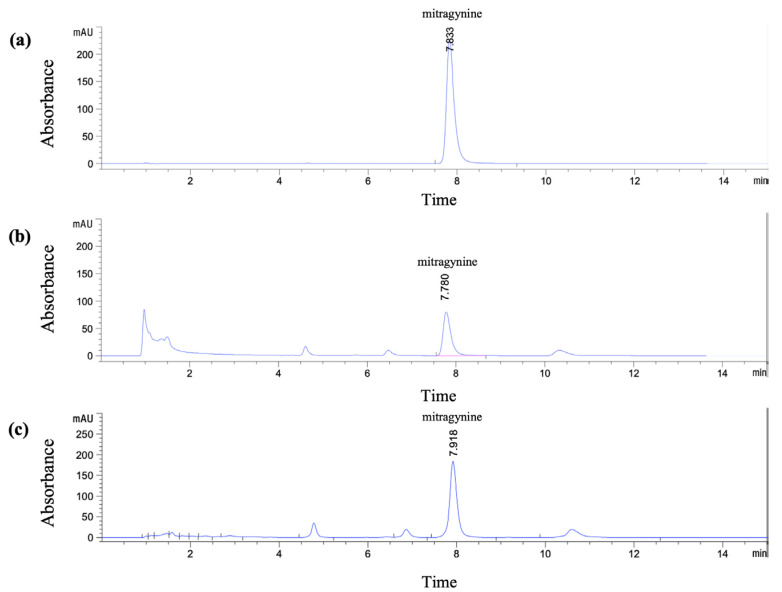
HPLC chromatograms of (**a**) standard mitragynine (100 µg/mL), (**b**) Kratom ethanolic extract (Et-MS, 0.5 mg/mL), and (**c**) alkaloid-rich extract (Alk-MS, 0.2 mg/mL). Analysis was performed using a Phenomenex^®^ C18 column (150 × 4.6 mm, 2.6 µm). The mobile phase consisted of 5.0 mM ammonium bicarbonate buffer and acetonitrile in a 40:60 (*v*/*v*) ratio, with a flow rate of 1.0 mL/min for 15 min.

The HPLC method for quantitative analysis of mitragynine in Kratom leaf powder and extracts was developed and validated with slight modifications from previously reported method [18,19]. The Kratom leaf powder used in this study contained 1.43% *w*/*w* mitragynine, as determined by HPLC. The crude ethanolic extract (Et-MS) was dark green in color, while the alkaloid-rich extract (Alk-MS) was dark brown. The extraction yields were 17.08% (*w*/*w*) for Et-MS and 15.37% (*w*/*w*) for Alk-MS. Mitragynine, selected as the marker compound, was identified by HPLC with a retention time of 7.83 min (Figure 1a). Mitragynine content was 4.96% *w*/*w* in Et-MS and 28.32% *w*/*w* in Alk-MS, calculated using the calibration equation y = 27.446x + 30.636 (R^2^ = 0.9994). A statistically significant difference (*p* < 0.05) was observed between the two extracts, with alkaloid-rich extract containing approximately five times more mitragynine than the ethanolic extract.

### 2.2. Antioxidant Profiles and Phytochemical Correlations of Kratom Extracts

The antioxidant activities of Et-MS, Alk-MS, and mitragynine were evaluated using DPPH, ABTS, and FRAP assays (Table 1). Et-MS showed the strongest antioxidant activity in the DPPH assay with an EC_5__0_ value of 0.06 ± 0.00 mg/mL, followed by Alk-MS (0.17 ± 0.03 mg/mL) and mitragynine (0.22 ± 0.01 mg/mL). A similar trend was observed in the FRAP assay, where Et-MS showed the highest reducing power (55.54 ± 1.23 g Fe^2+^/100 g sample), followed by mitragynine (36.30 ± 0.44) and Alk-MS (34.24 ± 0.95). Interestingly, in the ABTS assay, mitragynine demonstrated greater activity (EC_5__0_ = 0.16 ± 0.01 mg/mL) than both Et-MS (0.29 ± 0.03 mg/mL) and Alk-MS (0.42 ± 0.02 mg/mL), suggesting its ability to scavenge hydrophilic radicals such as ABTS•^+^. In terms of total phenolic content, Alk-MS contained the highest level (161.45 ± 4.90 mg GAE/g extract) followed by Et-MS (149.93 ± 2.16).

These results indicate that phenolic compounds play a major role in the antioxidant potential of Kratom leaf extracts. Although Alk-MS had higher mitragynine level, its antioxidant activity was lower than that of Et-MS in most assays, suggesting that antioxidant capacity depends not only on alkaloid concentration but also on the presence of phenolic and other redox-active constituents. This implies possible synergistic interactions among phenolic compounds, alkaloids, and other phytochemicals present in the crude extract [20,21].

The relatively high ABTS activity observed for mitragynine supports its intrinsic, non-phenolic antioxidant potential, likely related to its indole structure [13]. The antioxidant capacity of Kratom extracts appeared to be closely associated with their total phenolic content, consistent with previous reports showing a strong correlation between phenolic compounds and antioxidant activity in various plant species [20,21]. Together, these findings support the notion that whole-plant extracts, which retain a broader spectrum of phytochemicals, may offer superior bioactivity compared to purified compounds alone. This has important implications for the development of natural antioxidant or nutraceutical products based on Kratom extracts.

Although the Folin–Ciocalteu method provides an estimate of total reducing capacity rather than exclusively phenolic compounds, it serves as a useful comparative tool for assessing the overall antioxidant potential of plant extracts. Comprehensive LC–MS/MS profiling may be useful in future studies to clarify which phenolic and other antioxidant constituents are responsible for the observed activity.

### 2.3. Anti-Adipogenic Effects of Kratom Extracts and Mitragynine

#### Cell Viability

**Figure 2:**
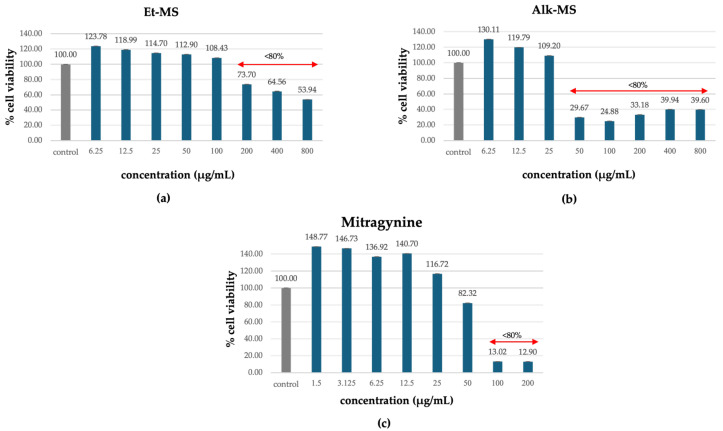
Cytotoxic effects of (**a**) Kratom ethanolic extract (Et-MS; 6.25–800 µg/mL), (**b**) alkaloid-rich extract (Alk-MS; 6.25–800 µg/mL), and (**c**) mitragynine (1.5–200 µg/mL) on 3T3-L1 cells, as determined by the MTT assay. Cell viability is expressed as a percentage relative to the untreated control group and presented as mean ± SEM (n = 3). A viability above 80% was considered non-cytotoxic and was used to determine test concentrations in subsequent assays.

3T3-L1 adipocytes are commonly used in adipogenesis assays due to their ability to carry out reproducible differentiation and their response to treatment [22,23,24]. Prior to assessing anti-adipogenic effects, cell viability was evaluated to identify non-cytotoxic concentrations of the extracts (Et-MS, Alk-MS) and mitragynine. Cells were treated with Et-MS and Alk-MS at concentrations ranging from 6.25 to 800 µg/mL and mitragynine concentrations ranging from 1.5 to 200 µg/mL. The MTT assay revealed that all three samples reduced cell viability in a concentration-dependent manner (Figure 2a–c). Concentrations that maintained ≥80% viability were considered non-cytotoxic and were selected for subsequent adipogenesis assays.

Notably, the alkaloid-rich extract (Alk-MS) and mitragynine exhibited higher cytotoxicity than the crude ethanolic extract (Et-MS), as reflected by reduced viability at lower concentrations. While Et-MS maintained high cell viability even at concentrations up to 200 µg/mL, Alk-MS and mitragynine showed a marked decline in viability at concentrations above 50 µg/mL. These findings are consistent with previous reports indicating that alkaloid fractions from *M. speciosa* contain more bioactive and potentially cytotoxic compounds, particularly mitragynine and its derivatives [13].

### 2.4. Inhibitory Effects on Lipid Accumulation During Pre-Adipocyte Differentiation

**Figure 3:**
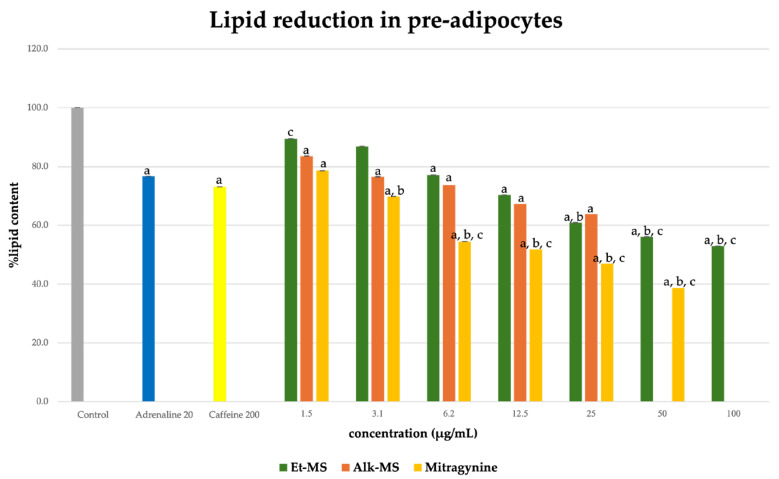
Lipid reduction by Et-MS, Alk-MS, and mitragynine in 3T3-L1 pre-adipocytes. Cells were treated during differentiation with Et-MS (1.5–100 µg/mL), Alk-MS (1.5–25 µg/mL), and mitragynine (1.5–50 µg/mL). Lipid content was quantified using Oil Red O staining on day 9. Results are expressed as mean ± SEM (n = 3). a *p* < 0.05 vs. control; b *p* < 0.05 vs. adrenaline (positive control); c *p* < 0.05 vs. caffeine (positive control).

**Figure 4:**
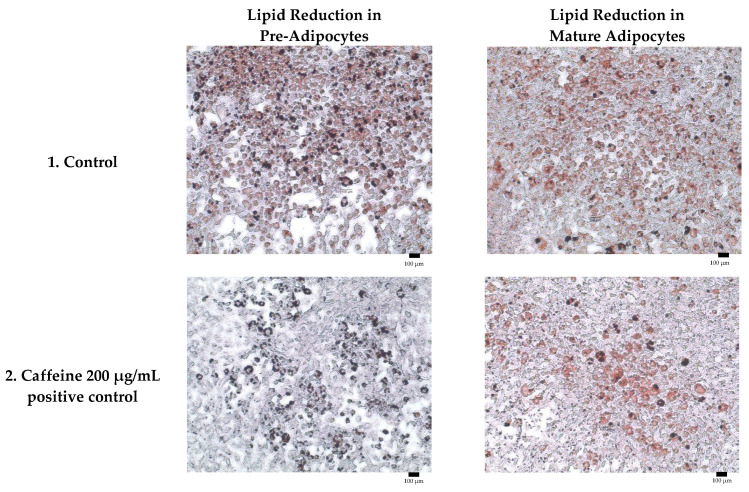

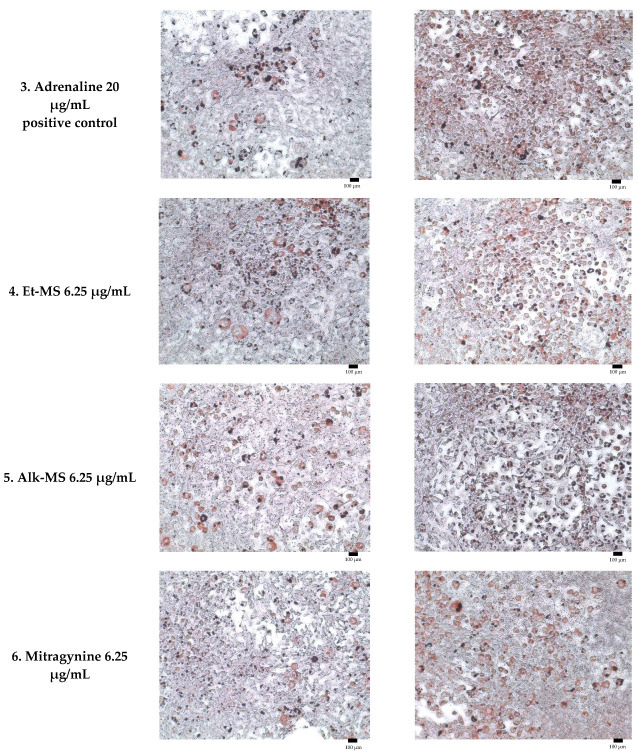
Representative micrographs of Oil Red O-stained 3T3-L1 adipocytes showing lipid reduction in both pre-adipocytes and mature adipocytes treated with Et-MS, Alk-MS, mitragynine, and positive controls. For lipid reduction in pre-adipocytes, extracts were applied on days 3, 5, and 7 after induction of differentiation and staining was performed on day 9. For lipid reduction in mature adipocytes, extracts were applied to mature adipocytes on day 9, and lipid accumulation was assessed on day 10. Positive controls included caffeine (200 µg/mL) and adrenaline (20 µg/mL). Images were captured at 10× magnification, and each panel includes a scale bar of 100 µm.

Adipogenesis is a complex, multistep process in which pre-adipocytes differentiate into lipid-filled adipocytes. Oil Red O staining is widely used to identify and quantify lipid accumulation, and it has become a standard method for assessing adipocyte differentiation in vitro [25]. As shown in Figure 3, all Kratom samples significantly inhibited lipid accumulation in a dose-dependent manner. Notably, Alk-MS and mitragynine exhibited anti-adipogenic effects stronger than Et-MS at lower concentrations. At 6.2–25 µg/mL, Alk-MS reduced lipid content to levels comparable to or significantly lower than the positive controls (*p* < 0.05). Similarly, mitragynine demonstrated robust activity across the 6.2–50 µg/mL range, suggesting it is the active principle contributing to the lipid-lowering effect. Among the extracts, Et-MS required higher concentrations (≥25 µg/mL) to achieve comparable inhibition. Although it showed weaker potency, Et-MS exhibited the lowest cytotoxicity, making it safer for long-term or topical applications. By comparison, adrenaline and caffeine reduced lipid accumulation by approximately 25–30% relative to the untreated control. However, both Alk-MS and mitragynine at lower concentrations (12.5–25 µg/mL) achieved significantly greater inhibition, with some treatments reducing lipid content by over 50%. This suggests that Kratom alkaloids may be more potent anti-adipogenic agents than the standard positive controls. Importantly, the potency–toxicity relationship must be considered. While Alk-MS and mitragynine showed superior efficacy, their cytotoxicity at higher concentrations (≥50 µg/mL) limits their safe therapeutic window (Figure 2a–c). In contrast, Et-MS maintained cell viability even at 100 µg/mL but required higher doses to achieve comparable lipid-lowering effects. This suggests that while alkaloid-rich fractions may exert stronger biological effects, including antioxidant and anti-adipogenic activities, their potential cytotoxicity must be carefully evaluated when considering dosage and delivery methods, especially for cosmeceutical applications. Microscopic images in Figure 4 show that treatment with Et-MS, Alk-MS, and mitragynine visibly reduced lipid droplet accumulation in both pre-adipocytes and mature adipocytes compared with the untreated control, consistent with the quantitative Oil Red O results. These morphological observations further confirm the inhibitory effects of the extracts and mitragynine on lipid accumulation during adipocyte differentiation, without observable cell damage.

Overall, these findings highlight a balance between potency and safety among the test samples. Mitragynine appears to be the key bioactive compound driving the anti-adipogenic activity of Kratom extracts. This is the first report to demonstrate the lipid-lowering effect of mitragynine in 3T3-L1 cells, supporting its relevance for anti-cellulite or fat-reducing cosmeceutical development.

### 2.5. Inhibitory Effects on Lipid Accumulation in Mature Adipocytes

**Figure 5:**
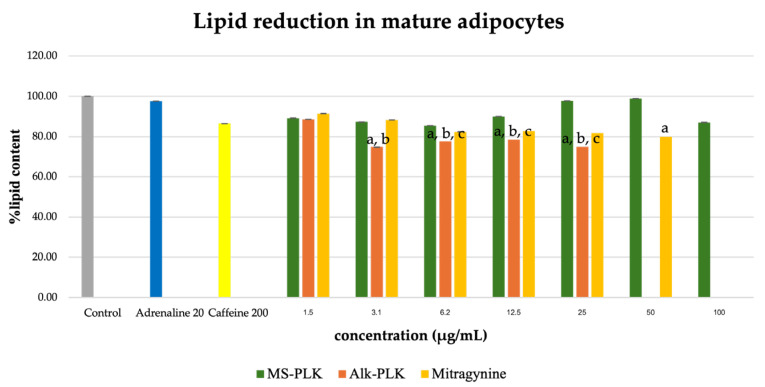
Lipid reduction by Et-MS, Alk-MS, and mitragynine in mature 3T3-L1 adipocytes. To assess lipid reduction in mature adipocytes, extracts were applied on day 9, and lipid content was measured on day 10. Et-MS was tested at concentrations of 1.5–100 µg/mL, Alk-MS at 1.5–25 µg/mL, and mitragynine at 1.5–50 µg/mL. Lipid content was quantified using Oil Red O staining. Results are expressed as mean ± SEM (n = 3). a *p* < 0.05 vs. control; b *p* < 0.05 vs. adrenaline (positive control); c *p* < 0.05 vs. caffeine (positive control).

The effect of the extracts on lipid accumulation in mature adipocytes was evaluated after treatment of differentiated 3T3-L1 cells. As shown in Figure 5, the ethanolic extract (Et-MS) showed minimal inhibition of lipid accumulation across the tested concentrations (1.5–100 µg/mL). In contrast, both the alkaloid-rich extract (Alk-MS) and mitragynine significantly reduced intracellular lipid levels in a concentration-dependent manner (*p* < 0.05). At 25 µg/mL, Alk-MS and mitragynine decreased lipid accumulation by approximately 25% and 18%, respectively, relative to the untreated control. The positive controls showed weaker responses under the same conditions: adrenaline (20 µg/mL) inhibited lipid accumulation by only ~2%, while caffeine (200 µg/mL) reduced it by ~14%. Although the degree of inhibition in mature adipocytes was modest (<30%), these results indicate that Kratom extracts and mitragynine retain measurable anti-adipogenic potential even at the late stage of adipocyte differentiation, outperforming standard positive controls such as caffeine.

## 3. Discussion

This study highlights the dual biological functions of Kratom extracts and its principal alkaloid, mitragynine, in modulating oxidative stress and adipogenesis, two key processes implicated in the pathophysiology of obesity and related dermal manifestations such as cellulite. The strong antioxidant activity demonstrated through DPPH, ABTS, and FRAP assays suggests that Kratom extracts can effectively scavenge reactive oxygen species (ROS), thereby mitigating oxidative stress [20,21,26] and potentially influencing transcription factors such as C/EBPβ and PPARγ, which play pivotal roles in adipocyte differentiation [14,15,16,27]. Previous studies have demonstrated that oxidative stress enhances adipogenesis and lipid accumulation through the activation of intracellular signaling pathways, with ROS specifically facilitating adipocyte differentiation by accelerating mitotic clonal expansion [28]. In contrast, antioxidants can inhibit adipocyte differentiation and lipid storage [11,12].

In this study, Kratom extracts, particularly the alkaloidal extract (Alk-MS) and mitragynine, significantly reduced lipid accumulation in 3T3-L1 cells, suggesting inhibition of adipogenic pathways. This lipid-lowering effect may also be linked to the modulation of lipolytic enzymes such as hormone sensitive lipase and pancreatic lipase, the latter of which has been shown to be inhibited by Kratom extracts in recent literature [10,29]. The dual activity of Kratom in reducing oxidative stress and inhibiting adipogenesis offers potential systemic benefits for metabolic syndrome and localized cosmetic concerns. By suppressing ROS and interfering with adipocyte maturation and lipid deposition, these extracts may attenuate subcutaneous fat accumulation and improve skin texture, thereby addressing aesthetic concerns like cellulite.

The observed anti-adipogenic effects of mitragynine and Kratom extracts may involve modulation of redox-sensitive signaling and energy-regulating pathways. Antioxidant compounds are known to influence adipogenesis by suppressing ROS and regulating the expression of key transcription factors such as PPARγ and C/EBPβ [27]. Mitragynine may exert similar effects through ROS reduction and potential activation of AMP-activated protein kinase (AMPK), which is well known to promote lipolysis and inhibit adipocyte differentiation [27,28]. These actions are consistent with mechanisms reported for other plant-derived compounds such as caffeine and resveratrol, which suppress adipocyte differentiation by enhancing AMPK activity and attenuating oxidative stress [30,31]. Such mechanistic parallels suggest that mitragynine and phenolic constituents in Kratom may act through comparable pathways to limit adipogenesis. In mature adipocytes, the lipid-reducing effect of Kratom extracts and mitragynine was statistically significant but modest (<30%). This result likely reflects the lower responsiveness of fully differentiated adipocytes and suggests that these compounds primarily act during the early phase of adipogenesis. Hence, while biologically meaningful, this inhibition should be interpreted cautiously in terms of clinical relevance.

From a cosmeceutical perspective, these properties are highly relevant. Antioxidants are widely used in skincare to combat oxidative damage and delay skin aging, while anti-adipogenic agents are increasingly incorporated into topical formulations aimed at cellulite reduction and body contouring. Both extracts and mitragynine showed no marked cytotoxicity at concentrations up to 25 µg/mL, supporting their potential safety for topical applications. However, the slightly higher cytotoxicity observed for the alkaloid-rich extract (Alk-MS) compared with pure mitragynine may result from the presence of other alkaloids or bioactive constituents that act additively or synergistically, a phenomenon commonly observed in crude herbal extracts. Considering the regulatory limitations associated with systemic use of Kratom extracts [9], a transdermal delivery route represents a safer and targeted strategy.

However, this study did not include molecular analyses of adipogenic markers such as PPARγ, C/EBPα, or FABP4, which are key regulators of lipid metabolism and adipocyte differentiation. Therefore, the proposed anti-adipogenic mechanisms are speculative and based on cellular lipid accumulation and antioxidant assays. Future studies should incorporate gene and protein expression analyses to confirm the specific molecular pathways underlying the observed effects.

In addition, this study was limited to in vitro assays and did not include percutaneous permeation studies or skin model experiments. Therefore, the results should be interpreted as preliminary evidence supporting the hypothesis that Kratom extracts and mitragynine possess cosmeceutical potential. Future work should focus on in vivo skin permeation, formulation optimization, and clinical evaluation to validate their efficacy in topical applications.

## 4. Materials and Methods

### 4.1. Chemicals and Reagents

The mitragynine standard used for quantitative analysis was obtained from ChromaDex^®^ (Irvine, CA, USA). All organic solvents employed in this study, including methanol, ethanol, acetonitrile, dichloromethane, and hexane, were of analytical or HPLC grade and were purchased from Merck (Darmstadt, Germany). Reagents used for phytochemical and antioxidant analyses, such as Folin–Ciocalteu reagent, sodium carbonate, and Trolox, were obtained from Sigma-Aldrich (St. Louis, MO, USA). All chemicals and reagents were of the highest purity available and were used without further purification. Deionized water was prepared using a Milli-Q water purification system (Millipore, Bedford, MA, USA) and used throughout the experiments.

### 4.2. Plant Material

The fresh leaves of Kratom were purchased from Phitsanulok provinces, Thailand. The plant material was authenticated by Dr.Pranee Nang-ngam, a botanist at the Faculty of Science, Naresuan University. A voucher specimen (Kaewchompoo001) was deposited in the herbarium of the Faculty of Science, Naresuan University. The leaves were washed with water, cut into pieces, and dried in a hot-air oven at 50–60 °C for 24 h. After drying, the materials were ground and blended to obtain a homogeneous powder. The Kratom leaf powder was stored in airtight containers at room temperature until use.

### 4.3. Extraction Process of Kratom Extract and Alkaloidal Extract

The Kratom leaf powder was macerated with 95% ethanol at a ratio of 1:10 (*w/v*) at room temperature for 1 h. This process was repeated three times, and the pooled filtrates were concentrated using a rotary evaporator at 45 °C to obtain the crude ethanolic extract (Et-MS).

The alkaloid-rich extract (Alk-MS) was prepared following the methods described by Jamil et al. [32] and Shamima et al. [33], with slight modifications. Briefly, Et-MS were dissolved in ethanol, and 10% acetic acid was added to acidify the solution. The acidic aqueous layer was then partitioned with hexane to remove non-polar components. The remaining aqueous phase was basified with sodium carbonate and stirred until the pH reached 9–11, during which the solution turned grayish brown. To extract the alkaloids, dichloromethane was added, and the mixture was transferred to a separating funnel. The organic layer was collected and filtered. The dichloromethane was then evaporated under reduced pressure using a rotary evaporator until dryness, yielding Alk-MS.

### 4.4. Determination of Mitragynine by HPLC Technique

The HPLC analysis was performed using an isocratic method based on Thai herbal pharmacopeia [18] and Mudge et al. [19] with slight modifications. The HPLC system (Agilent^®^ 1260; Agilent Technologies, USA) was equipped with a DAD detector (G7129A 1260 vial sampler, G711A 1260 Quat Pump VL and G7115A 1260 DAD). The separation was carried out using a C18 column (Phenomenex^®^, 150 × 4.6 mm, 2.6 µm) maintained at 30 °C. The mobile phase consisted of 5.0 mM ammonium bicarbonate buffer (A) and acetonitrile (B) in a ratio of 40:60, delivered at a flow rate of 1 mL/min for 15 min. Kratom leaf powder (50 mg) was extracted with methanol (10 mL), while the extracts were dissolved in methanol at final concentrations of 0.5 mg/mL for Et-MS and 0.2 mg/mL for Alk-MS. A volume of 5 µL was injected into the HPLC system and detection was performed at 226 nm. Mitragynine content was quantified using a linear regression equation derived from a standard calibration curve. The method was validated according to ICH Q2(R1) guidelines. Linearity was confirmed in the range of 2.5–100 µg/mL with a correlation coefficient (R^2^) of 0.9994. The LOD and LOQ for mitragynine were 0.25 µg/mL and 2.5 µg/mL, respectively. Intra- and inter-day precision (RSD) were below 2.0%, and recovery ranged from 94.8% to 98.2%, demonstrating the accuracy, precision, and reliability of the analytical method.

### 4.5. Total Phenolic Content

The phenolic content of Et-MS and Alk-MS was determined using the Folin–Ciocalteu method [22], with gallic acid as the standard. Extracts and gallic acid standard solutions were prepared in 70% methanol at 1 mg/mL. A calibration curve was generated by two-fold serial dilutions of gallic acid (6.25–200 µg/mL). A 25 µL aliquot of either the extract or standard was mixed with 25 µL of tenfold-diluted Folin–Ciocalteu reagent and 200 µL of distilled water. After 5 min, 25 µL of sodium carbonate solution was added, and the mixture was incubated at room temperature in the dark for 60 min. The absorbance was measured at 725 nm using a microplate reader (BioTek, Winooski, VT, USA). All determinations were performed in triplicate. The results were expressed as milligrams of gallic acid equivalents per gram of extract (mg GAE/g extract).

### 4.6. Determination of Antioxidant Activity

#### 4.6.1. DPPH Radical-Scavenging Assay

The antioxidant activity was determined using a DPPH radical scavenging assay following the methods of Goh et al. [13] and Supakit et al. [34] with slight modifications. Ascorbic acid (positive control) and test samples were dissolved in methanol at concentrations ranging from 0.0001 to 3 mg/mL and mixed with 150 µL of 0.2 mM DPPH solution. After 30 min of incubation at room temperature in the dark, absorbance was measured at 515 nm using a microplate reader. A solution of 150 µL DPPH and 75 µL methanol served as the control. All tests were performed in triplicate, and EC_5__0_ values were calculated and reported as mean ± SD. The radical scavenging activity (RSA) was calculated using the following equation:

%RSA = ((A_control − A_sample)/A_control) × 100

where A represents the absorbance.

#### 4.6.2. ABTS Antioxidant Assay

The Trolox equivalent antioxidant capacity (TEAC) was determined using the ABTS assay, according to the method of Goh et al. [13] and Pinchuk et al. [35] with minor modifications. ABTS was generated by reacting 7 mM ABTS with 2.45 mM potassium persulfate in water and allowing the solution to stand in the dark at room temperature for 12–16 h. The resulting ABTS solution was then diluted with water to an absorbance of 0.70 ± 0.02 at 734 nm. A 20 µL aliquot of the sample or Trolox (positive control; 0.0001–3 mg/mL) was added to 180 µL of the ABTS solution. After 6 min of incubation, the absorbance was measured at 734 nm. %RSA was calculated using the equation mentioned above. All tests were performed in triplicate, and EC_5__0_ values were calculated and reported as mean ± SD.

#### 4.6.3. FRAP (Ferric-Reducing Antioxidant Power) Assay

The reducing capacity of the extracts was determined using the FRAP assay as described by Goh et al. [13] with slight modifications. The FRAP reagent was prepared by mixing 300 mM acetate buffer (pH 3.6), 10 mM TPTZ (2,4,6-Tris(2-pyridyl)-s-triazine), and 20 mM ferric chloride in a ratio of 10:1:1. A 20 µL aliquot of the sample (1 mg/mL in methanol) or FeSO4.7H2O standard solution (0.01–1 mM) was added to 180 µL of the FRAP reagent. After 30 min of incubation at room temperature in the dark, absorbance was measured at 595 nm using a microplate reader. All determinations were performed in triplicate. The FRAP values were expressed as grams of Fe^2+^ equivalents per 100 g of sample.

### 4.7. Evaluation of Cytotoxicity and Anti-Adipogenic Activity in 3T3-L1 Cells

#### 4.7.1. Cell Line and Culture

The experimental protocol for the use of 3T3-L1 preadipocytes and adipocytes was approved by Naresuan University (Approval number: NUIBC OT 67-01-08). The 3T3-L1 cell line was obtained from the JCRB Cell Bank (Japan). Cells were cultured in Dulbecco’s Modified Eagle Medium (DMEM) supplemented with 10% calf serum, 3.7 g/L sodium bicarbonate, and 100 U/mL penicillin-streptomycin. The cultures were maintained at 37 °C in a humidified incubator with 5% CO^2^. Cells were sub-cultured twice weekly using trypsin/EDTA solution in 175 cm^2^ culture flasks [25,26].

#### 4.7.2. Cytotoxicity Assay

The cytotoxic effects of extracts were assessed using the MTT assay. The experiment was conducted following the protocols reported by Tohar et al. and Ngamdokmai et al. [27] with minor modifications. Cells were harvested at 70–80% confluency and centrifuged at 1200 rpm at 25 °C for 5 min. The cells were seeded at a density of 1×10^4^ cells per well in a 96-well plate and incubated in 5% CO_2_ for 24 h to allow cell attachment. After incubation, the culture medium was carefully removed. The extracts were dissolved in 100% dimethyl sulfoxide (DMSO) and diluted into the culture medium to obtain final concentrations ranging from 7.81 to 800 µg/mL. The final concentration of DMSO in the cell culture medium was kept below 1% *v*/*v*. Then, 50 µL of MTT working solution (1 mg/mL in phosphate-buffered saline [PBS], pH 7.4) was added to each well and incubated for an additional 3 h. After incubation, the unreacted dye was removed, and the resulting insoluble formazan crystals were dissolved in 100 µL DMSO per well. The absorbance was measured at 595 nm using a microplate reader. Cell viability was calculated using the following equation [29,30].

% Viability = (Absorbance_595_ nm of sample/Absorbance_595_ nm of control) × 100

The absorbance of the untreated control group was considered 100%, and the viability of all other treatment groups was expressed as a percentage relative to this control.

#### 4.7.3. Determination of Lipid Accumulation by Oil Red O Staining

T3-L1 Differentiation and Extract Treatment

The determination of lipid accumulation by Oil Red O staining was carried out following the protocol reported by Ngamdokmai et al. [24] with slight modifications. 3T3-L1 preadipocytes were seeded to confluence in a 96-well plate and cultured in Dulbecco’s Modified Eagle’s Medium (DMEM) containing 10% calf serum, 3.7 g/L sodium bicarbonate, and 100 U/mL penicillin-streptomycin. The culture was maintained at 37 °C in a humidified incubator with 5% CO_2_. Differentiation was induced using DMEM supplemented with 10% fetal bovine serum (FBS), 1 µM dexamethasone (DEX), 0.5 mM isobutyl methylxanthine (IBMX), and 10 µg/mL insulin. After 48 h, the medium was replaced with DMEM containing 10% FBS and 10 µg/mL insulin, which was refreshed every 48 h for a total of 9 days [22,23,24,36,37]. In this study, 3T3-L1 pre-adipocytes were induced to differentiate using a cocktail of dexamethasone, 3-isobutyl-1-methyl xanthine (IBMX), and insulin. Differentiation progressed over 9 days, at which point mature adipocytes were evaluated by Oil Red O staining. The lipid reduction in pre-adipocytes and mature adipocytes of Et-MS, Alk-MS and mitragynine on adipogenesis were assessed at non-cytotoxic concentrations. To evaluate lipid reduction in pre-adipocytes, the test samples were added to the culture medium on days 3, 5, and 7 during differentiation. During this period, the media combined with extracts was replaced every 2 days for a total of three treatments. Lipid accumulation was quantified on day 9. For lipid reduction in mature adipocytes, mature adipocytes were treated on day 9, and lipid content was measured on day 10. Positive controls included caffeine (200 µg/mL) and adrenaline (20 µg/mL), known anti-adipogenic agents.

Oil Red O staining

After adipogenesis, lipid droplets were visualized through Oil Red O staining of 3T3-L1 adipocytes treated with different concentrations of the extracts for 24 h. The cells were washed twice with PBS, then fixed with 10% formalin for 8 min and again for 1 h at room temperature. Subsequently, cells were washed with 60% isopropanol and stained with freshly prepared Oil Red O solution (prepared by mixing 3 parts of 0.5% Oil Red O stock solution with 2 parts distilled water) for 45 min at room temperature. The cells were then washed with double-distilled water to remove excess stain and air-dried. Stained lipid droplets were examined under a microscope. Oil Red O was extracted using isopropanol after 10 min, and absorbance was measured at 520 nm using a microplate reader [22,23,24,36,37].

### 4.8. Statistical Analysis

The antioxidant activity, total phenolic content, and mitragynine content (%*w*/*w*) were investigated in independent triplicate experiments. Data were expressed as the mean ± standard deviation (SD). In vitro data were expressed as the mean ± standard error of the mean (SEM), based on triplicate measurements, using GraphPad Prism software (version 10). Statistical significance was determined using one-way analysis of variance (ANOVA), with *p* < 0.05 considered statistically significant.

## 5. Conclusions

This study demonstrated that Kratom extracts and its major alkaloid, mitragynine, exhibit both antioxidant and anti-adipogenic activities that support their potential application in cosmeceutical formulations. The ethanolic extract (Et-MS) showed potent antioxidant capacity with DPPH and ABTS EC_5__0_ values of 0.06 mg/mL and 0.29 mg/mL, respectively, while the alkaloid-rich extract (Alk-MS) displayed slightly lower activity. In 3T3-L1 adipocytes, Alk-MS and mitragynine reduced lipid accumulation by approximately 50–70% at non-cytotoxic concentrations (≤25 µg/mL), whereas Et-MS required higher doses to achieve comparable inhibition. These results suggest that phenolic constituents mainly contribute to antioxidant capacity, whereas mitragynine is the principal anti-adipogenic compound. Together, these findings provide preliminary in vitro evidence supporting the development of Kratom-derived ingredients for anti-cellulite and antioxidant cosmeceutical applications.

## Figures and Tables

**Table 1:** Antioxidant activities determined by DPPH, ABTS, and FRAP assays, total phenolic content, and mitragynine content (% *w*/*w*) of Kratom ethanolic (Et-MS) and alkaloid-rich (Alk-MS) extracts.

Sample/ Positive Control	DPPH EC_5__0_ (mg/mL)	ABTS EC_5__0_ (mg/mL)	FRAP (g Fe^2+^/100 g)	Total Phenolic (mg GAE/g)	% *w*/*w* Mitragynine
Et-MS	0.06 ± 0.00 ^a^	0.29 ± 0.03 ^a^	55.54 ± 1.23 ^a^	149.93 ± 2.16 ^a^	4.96 ± 0.12 ^b^
Alk-MS	0.17 ± 0.03 ^b^	0.42 ± 0.02 ^b^	34.24 ± 0.95 ^b^	161.45 ± 4.90 ^b^	28.32 ± 0.37 ^a^
Mitragynine	0.22 ± 0.01 ^c^	0.16 ± 0.01 ^c^	36.30 ± 0.44 ^b^	NA	NA
Ascorbic acid	0.002 ± 0.00 ^d^	ND	ND	ND	NA
Trolox	ND	0.04 ± 0.00 ^d^	ND	ND	NA

Values are expressed as mean ± SD (n = 3). Different superscript letters (a, b, c, d) within each column indicate significant differences (*p* < 0.05) as determined by one-way ANOVA. ND = Not Determined; NA = Not Applicable.

## Data Availability

Data is contained within the article.
